# Supplementary material for: Rumen Microbiota Predicts Feed Efficiency of Primiparous Nordic Red Dairy Cows
Source: Microorganisms. 2023 Apr 25;11(5):1116. doi: 10.3390/microorganisms11051116 (PMC10222030; doi:10.3390/microorganisms11051116)
Supplement: Supplementary file 1 [file microorganisms-11-01116-s001.zip › microorganisms-2334079-supplementary.pdf]

Table S1. Mean (± standard deviation) composition of the experimental feeds during years 2018 - 2020.

	Grass silage	Concentrate mix	
		Feeding station	Milking parlor[1]
n	46	41	41
Dry matter (DM), g/kg	271.3 ± 34.9	878.0 ± 6.2	878.2 ± 6.8
In DM, g/kg			
Ash	80.1 ± 12.6	73.1 ± 2.4	75.1 ± 2.9
Crude protein	149.1 ± 14.8	205.2 ± 14.8	211.1 ± 13.6
Crude fat		35.7 ± 7.7	30.1 ± 5.9
NDF	519.8 ± 42.1	232.7± 10.4	217.1 ± 14.2
Fermentation quality, g/kg DM			
pH	4.13 ± 0.20		
Ammonia N, g/kg N	36.6 ± 9.3		
Lactic acid	50.5 ± 23.4		
Acetic acid	18.1 ± 3.9		
Propionic acid	1.26 ± 1.3		
Butyric acid	0.42 ± 0.67		
D-value[2], g/kg DM	681 ± 32.5		
ME[3], MJ/kg DM	10.9 ± 0.52	12.0 ± 0.10	12.1 ± 0.16

[1]Milking parlor concentrate mix was given 600 g/d.

[2]In vitro digestible organic matter, g/kg dry matter.

[3]Metabolizable energy.

Table S2. Mean (± standard deviation) feed and nutrient intake from calving to rumen sampling of the cows sampled during the different years.

	Sampling year		
	2018	2019	2020
n	21	28	38
Intake			
Dry matter, kg/d	19.5 ± 1.25	21.1 ± 1.88	19.7 ± 1.39
Grass silage, kg DM/d	9.8 ± 0.72	11.2 ± 1.66	9.9 ± 0.99
Concentrate, kg DM/d	9.7 ± 0.59	9.9 ± 0.45	9.8 ± 0.51
Crude protein, kg/d	3.51 ± 0.21	3.79 ± 0.30	3.45 ± 0.22
NDF[1], kg/d	7.34 ± 0.50	7.70 ± 0.82	7.72 ± 0.66
ME[2], MJ/d	214.4 ± 11.90	227.0 ± 17.91	214.1 ± 13.12

[1]Neutral detergent fiber.

[2]Metabolizable energy.

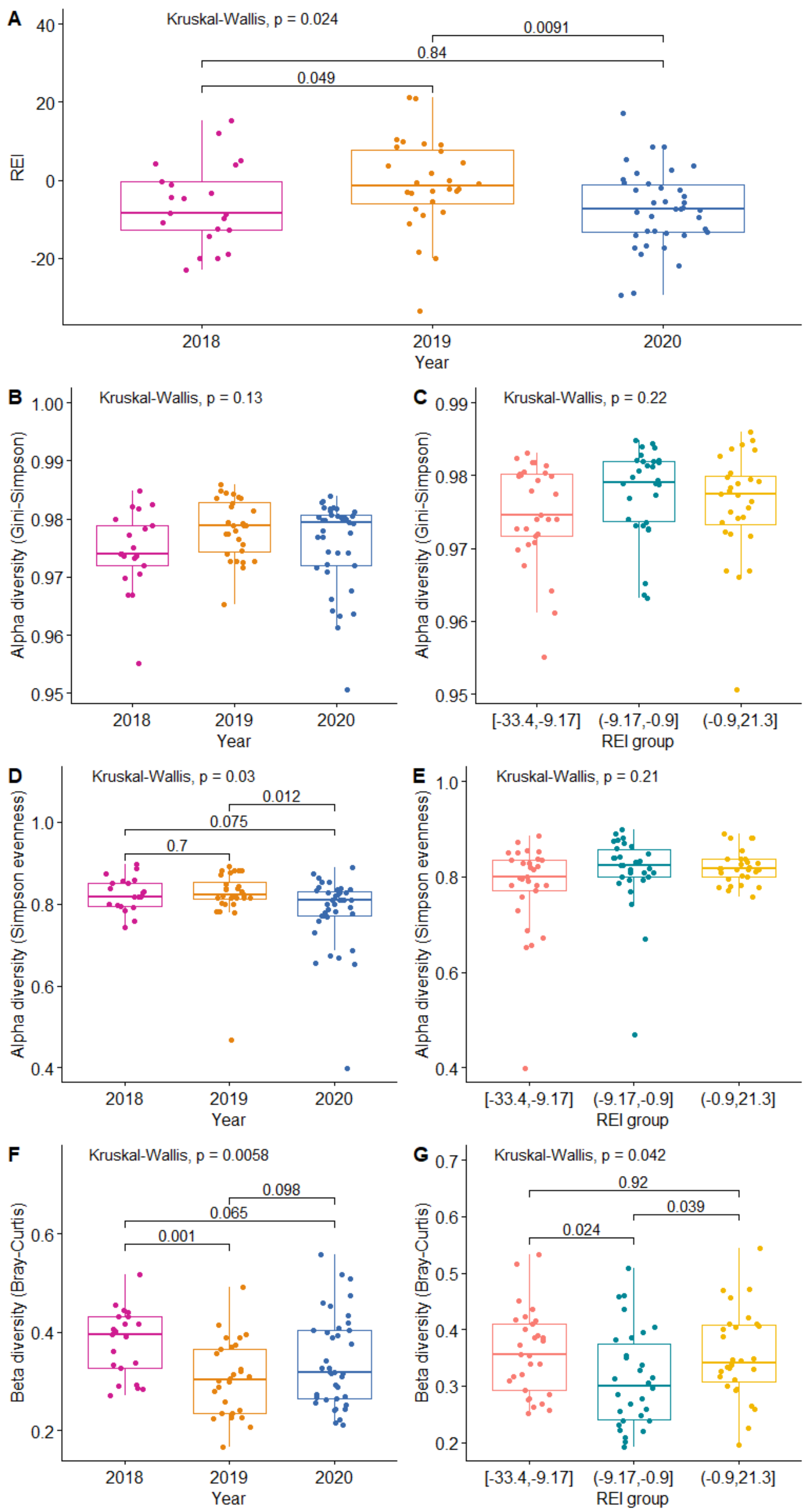

Figure S1. Box plots of feed efficiency and diversity per year and per REI group. A) REI, and B) Gini-Simpson, D) Simpson Evenness and F) Bray-Curtis beta diversity per year, and the diversities per REI groups in C, E, and G, respectively. Kruskal-Wallis test for the group effect and pairwise Wilcoxon test estimates indicated in the sub-figures.

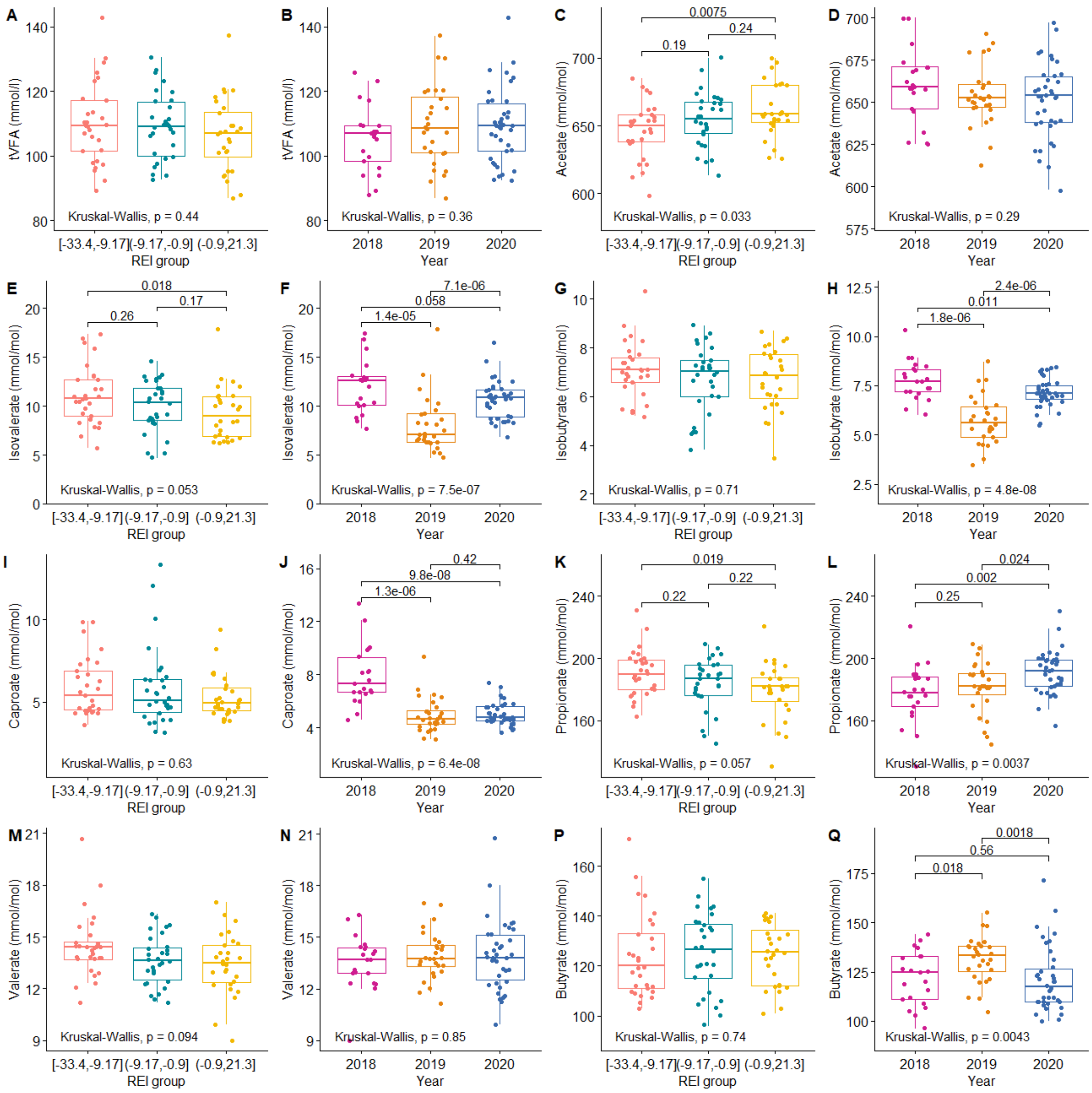

Figure S2. Box plot of volatile fatty acid data per year and per REI group. Total Volatile Fatty Acids (tVFA), Acetate, Isovalerate, Isobutyrate, Caproate, Propionate, Valerate, and Butyrate grouped according to REI tertiles and by year. Kruskal-Wallis test for the group effect and pairwise Wilcoxon test estimates indicated in the sub-figures.

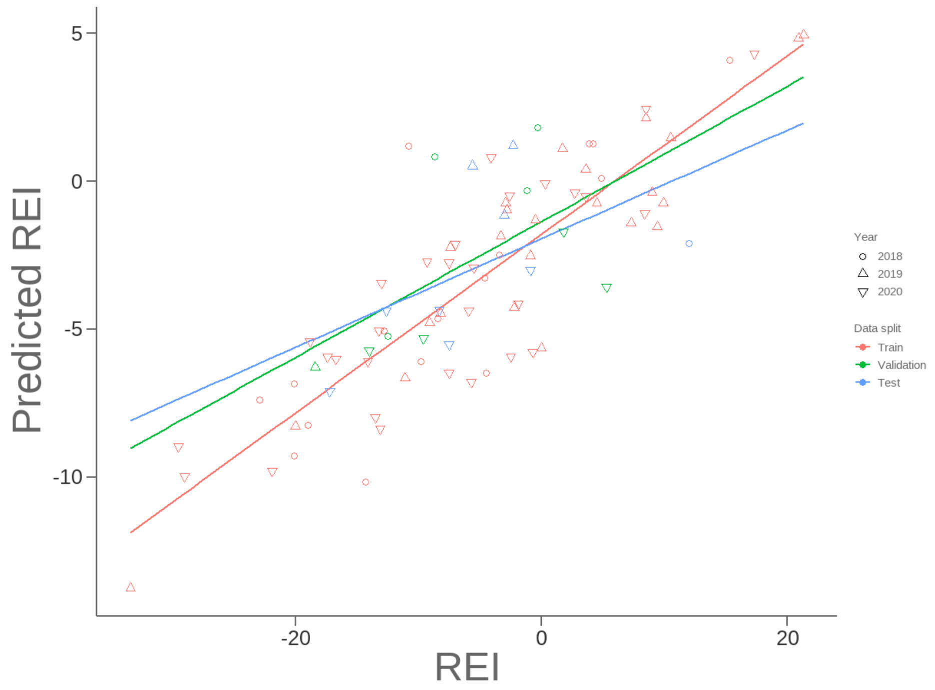

Figure S3. Correlation between phenotypically estimated Residual Energy Intake on the first 100 days in milk (REI) and the predicted REI values from the model. Correlations for train, validation and test data were 0.88, 0.60, and 0.55, respectively. The samples from different years do not show any systematic bias.

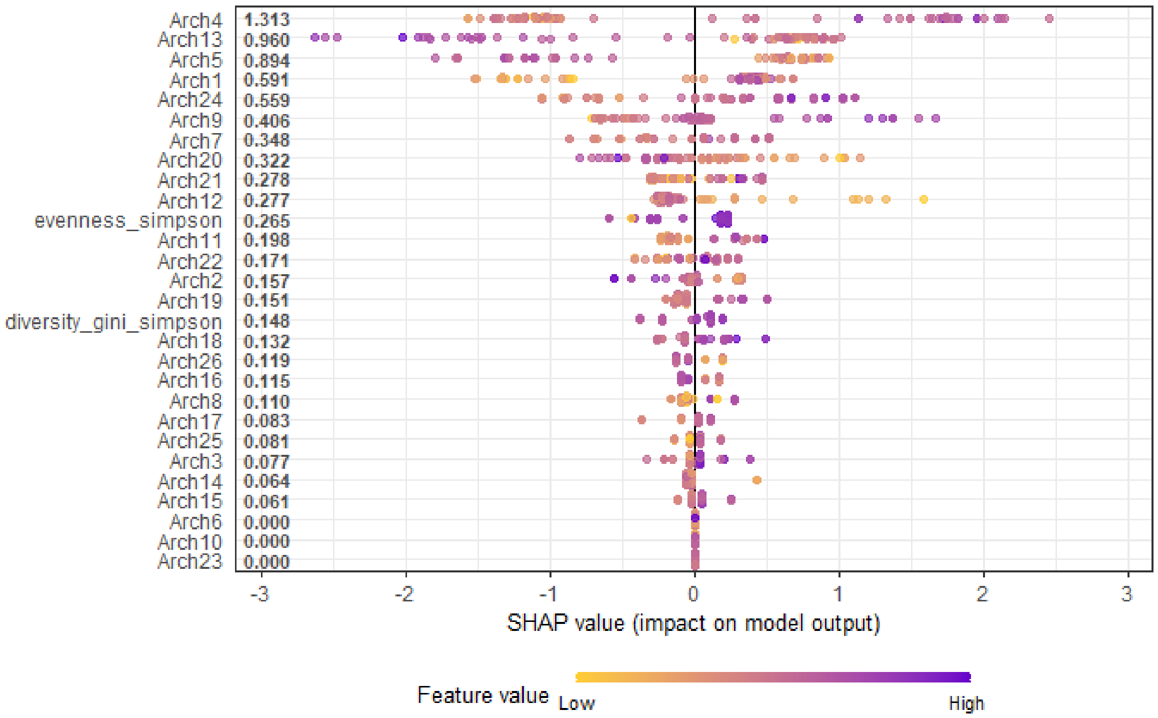

Figure S4. Shapley summary plot with predictors sorted in SHAP importance order from the most important (top) to least important (bottom). The X- axis indicates predictor impact in each sample, with coloring indicating low or high predictor values. The feature importance given next to the y-axis.

A

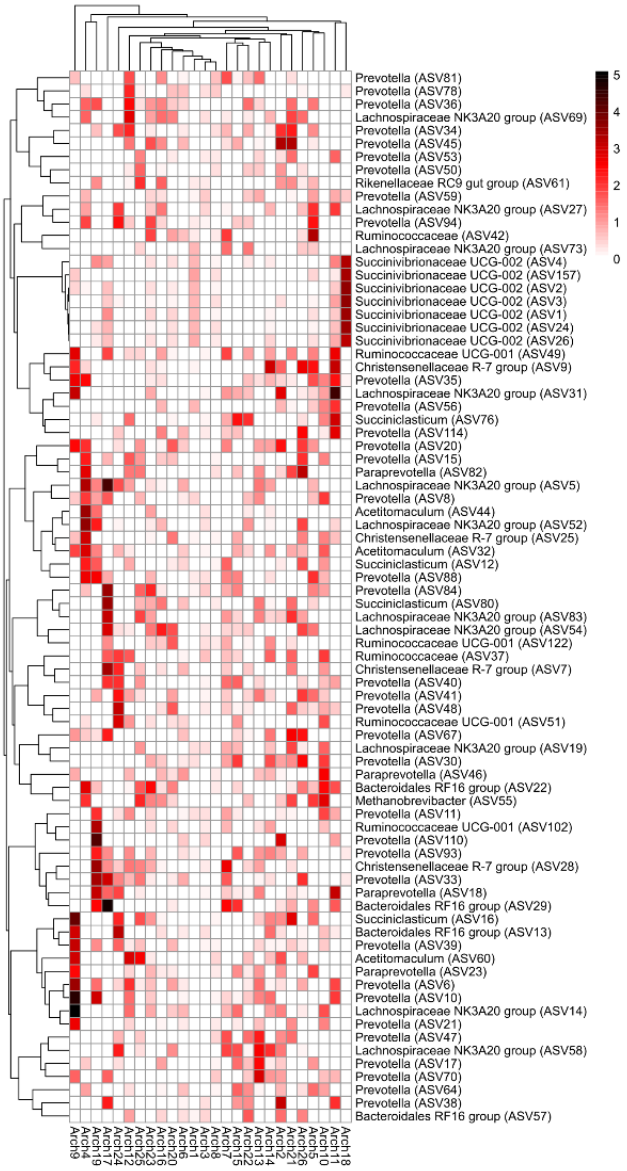

B

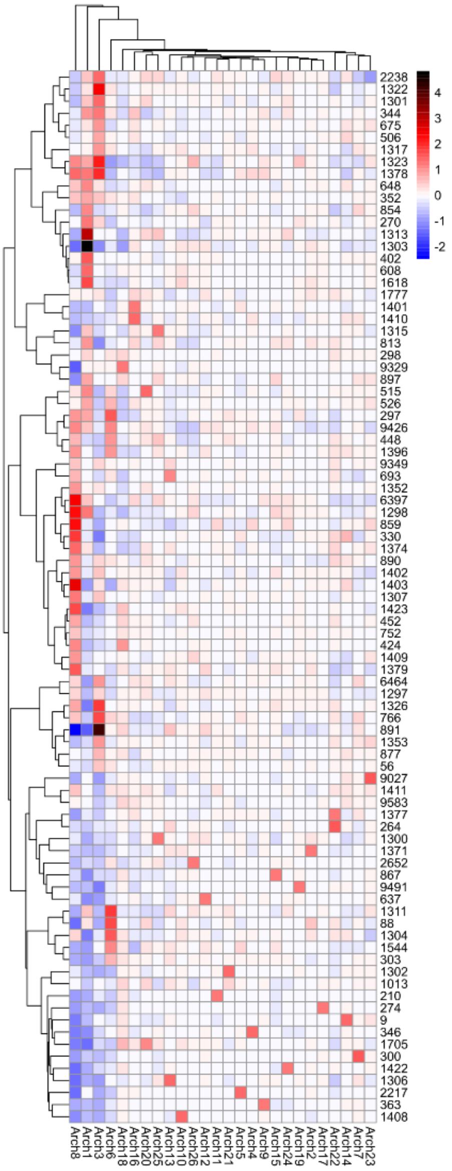

Figure S5. Heat maps for A) transposed Y matrix of the GLRM analysis indicating ASV abundancy profile represented by each of the archetype components and for B) the X matrix of the GLRM analysis indicating archetype scores for each cow sample.

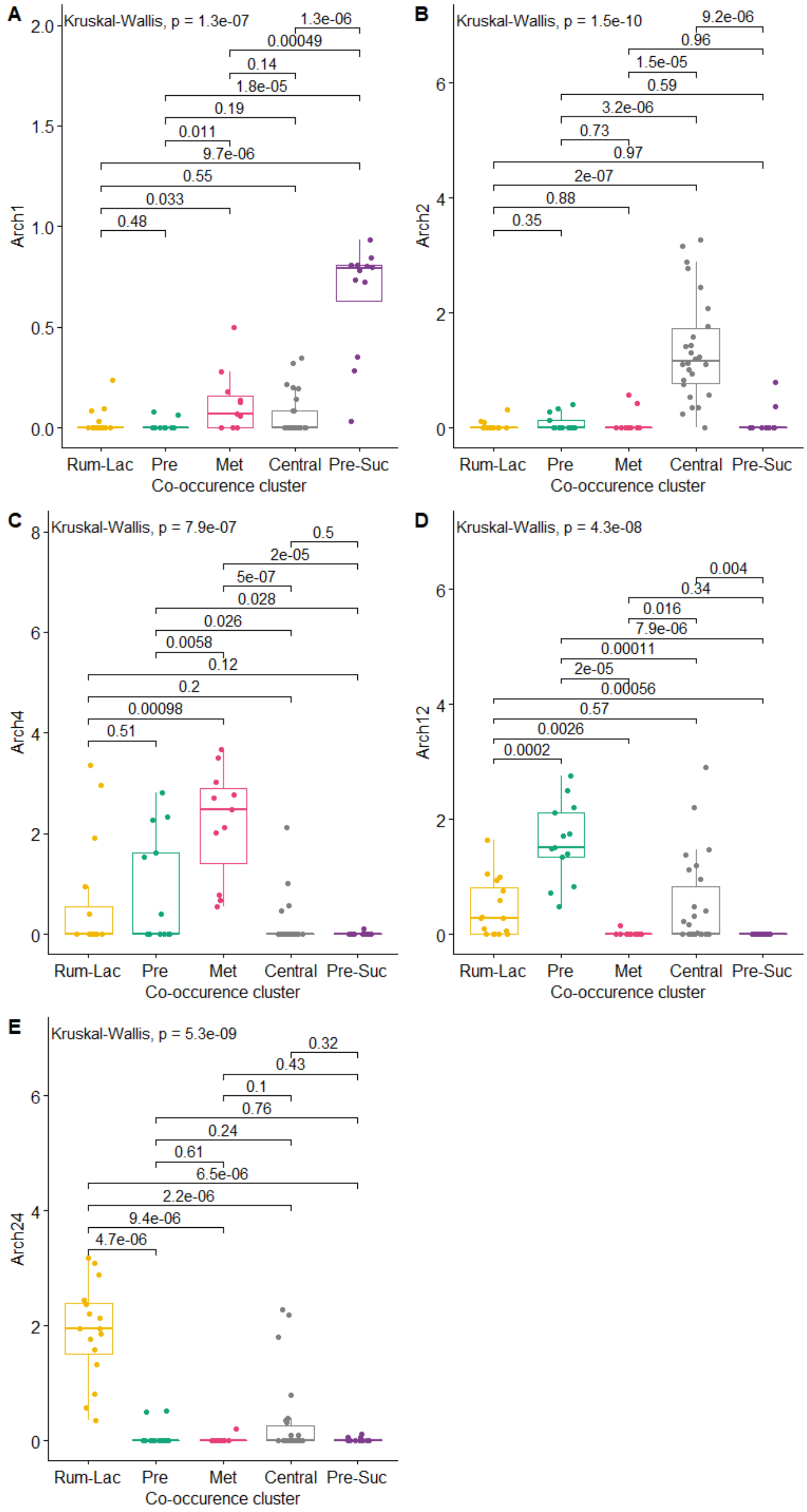

Figure S6. Box plot for values of archetype components Arch 1, 2, 4,12 and 24 (A to E, respectively) values for ASVs in the five co-occurrence clusters.

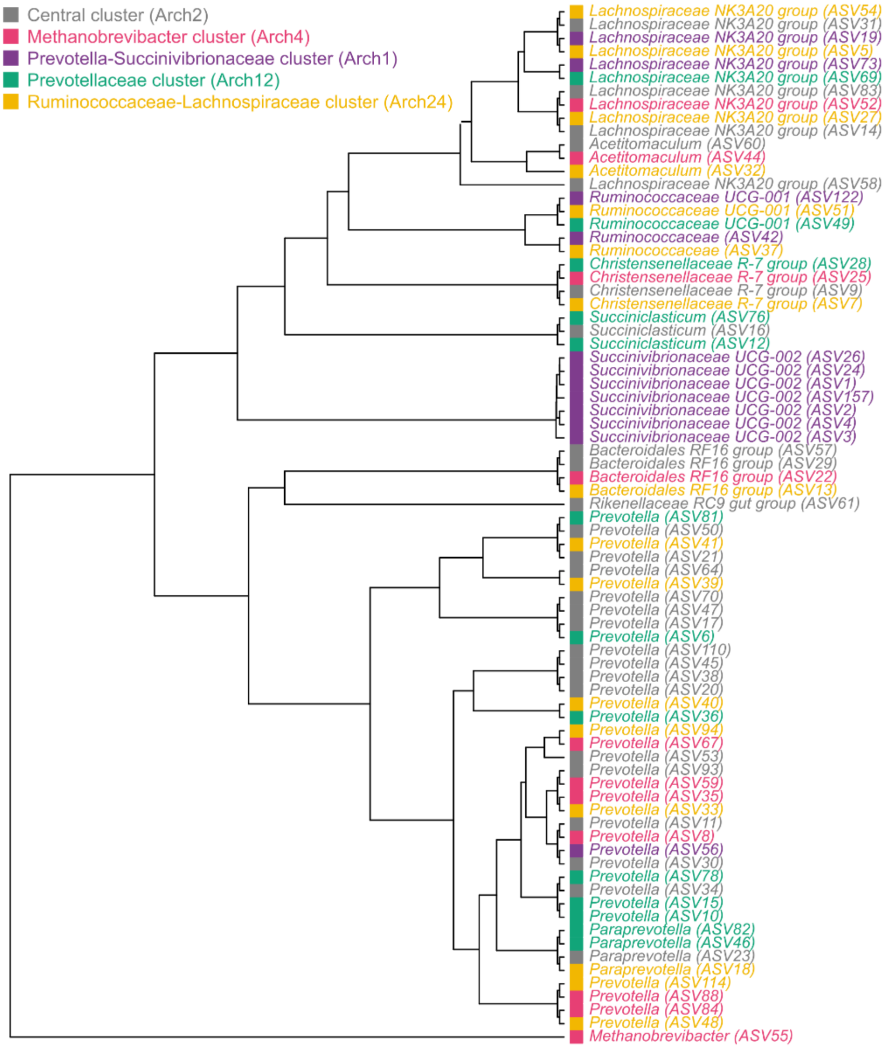

Figure S7. Phylogenetic tree of 16S rRNA amplicon based ASVs annotated with the taxonomical names. The colored groups match the modules of the co-occurrence network in Figure 5.

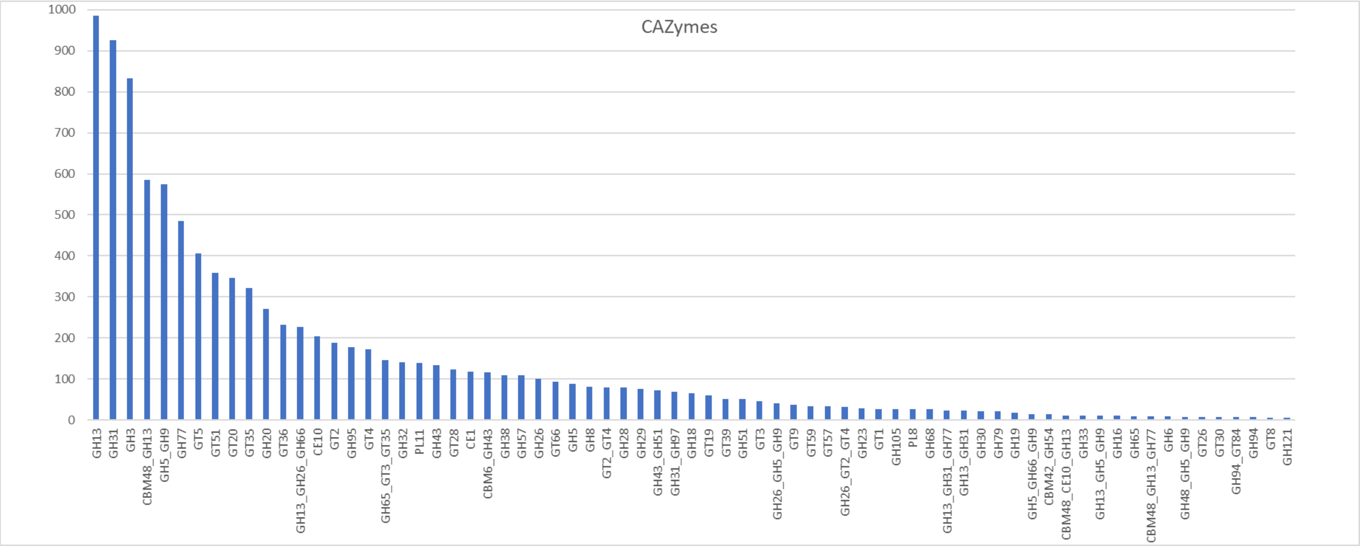

Figure S8. Bar chart presenting the abundances of CAZy families in the total data set, calculated as the average abundance across all samples and sorted from to most abundant to the least abundant.

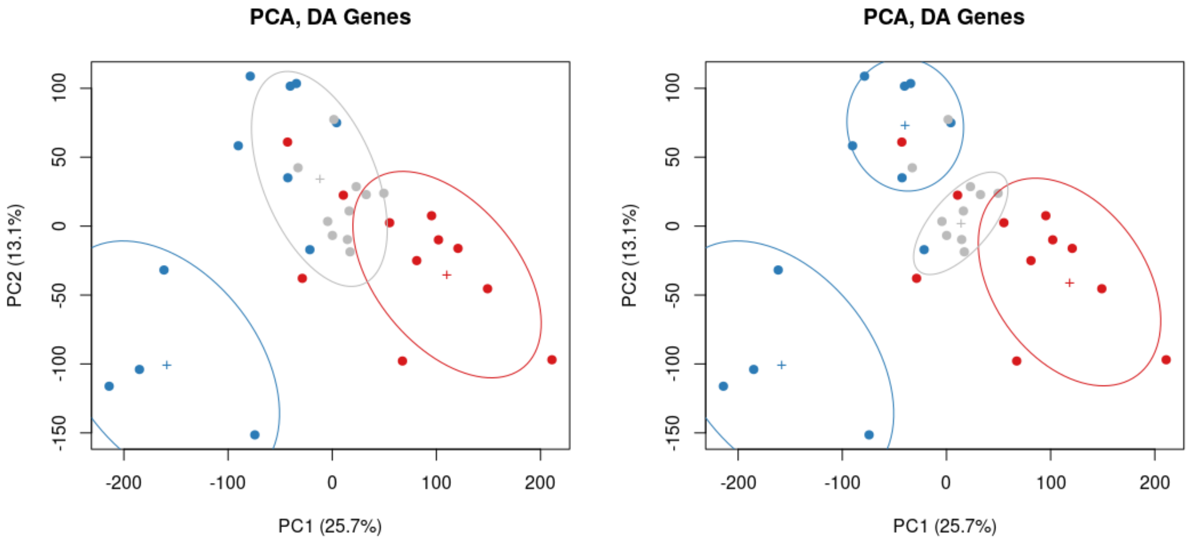

Figure S9. Scatterplot of first two principal components for the differentially abundant (DA) genes. Explained variance per component is indicated in the axes-labels. Red dots indicate L-REI samples, blue ones H-REI samples and the gray ones the intermediate samples. Two different k-mean classifications have been applied, k=3 (left) and k=4 (right) to indicate possible groupings. Ellipses represent the covariance matrices of each cluster with respect to their center, indicated with '+'.

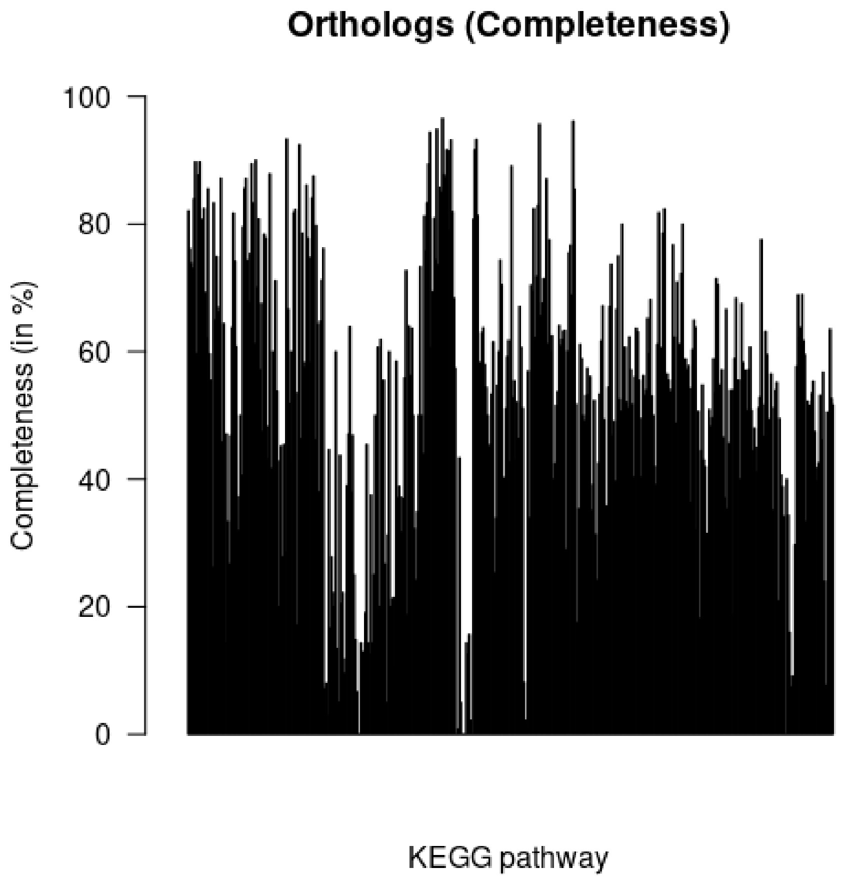

Figure S10. Completeness of detected KEGG pathways. KEGG pathways are sorted on the x-axis, and y-axis indicates the completeness between 0 and 100%, based on identified KEGG Orthologs from in-silico gene predictions and EggNOG annotations.

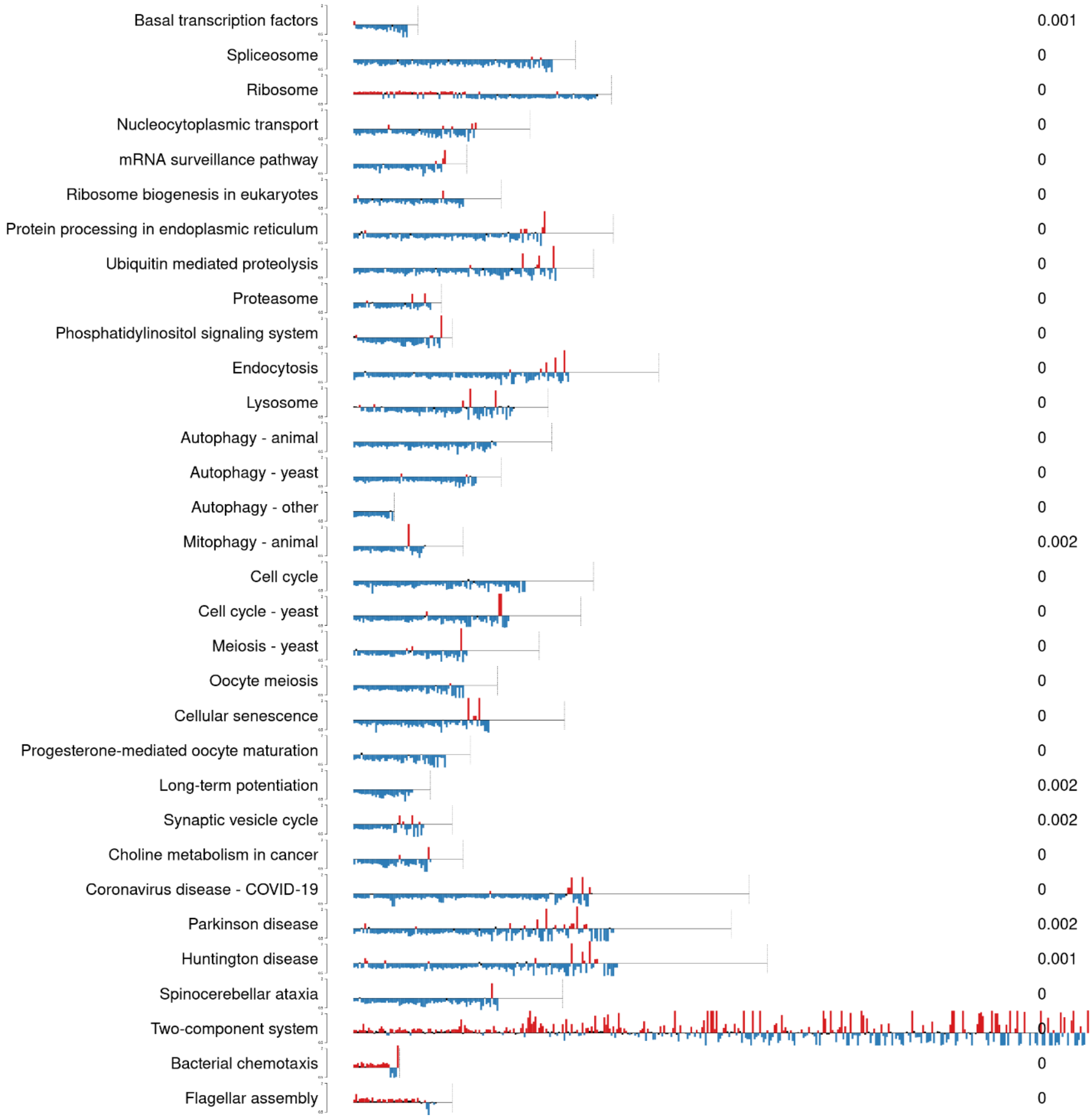

Figure S11. Visualization of the KO-wise abundance ratio between the L-REI (red) and H-REI (blue) groups within each significant pathway. Each row represents a pathway, each bar a KO, the line represents the total set of pathway orthologs showing here also the detected completeness of tested pathways. The p-values given in the last column. Y-axes are all fixed between 0.5 and 2.

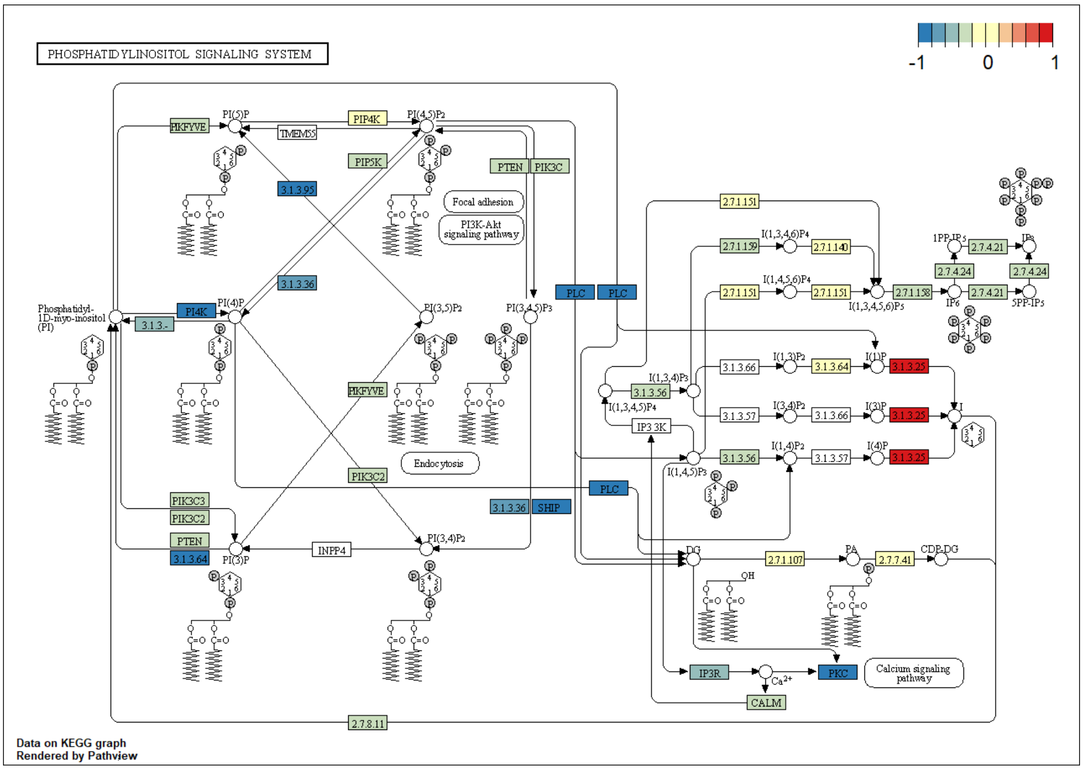

Figure S12. Representation of the metagenomic gene abundance data for Phosphatidylinositol signaling system pathway (ko04070). KO's (orthologs) more abundant among L-REI animals are in red and genes more frequent in H-REI are in blue, respectively. Values outside the range –1 to 1 truncated within these boundaries. KO's missing in the current data are white.

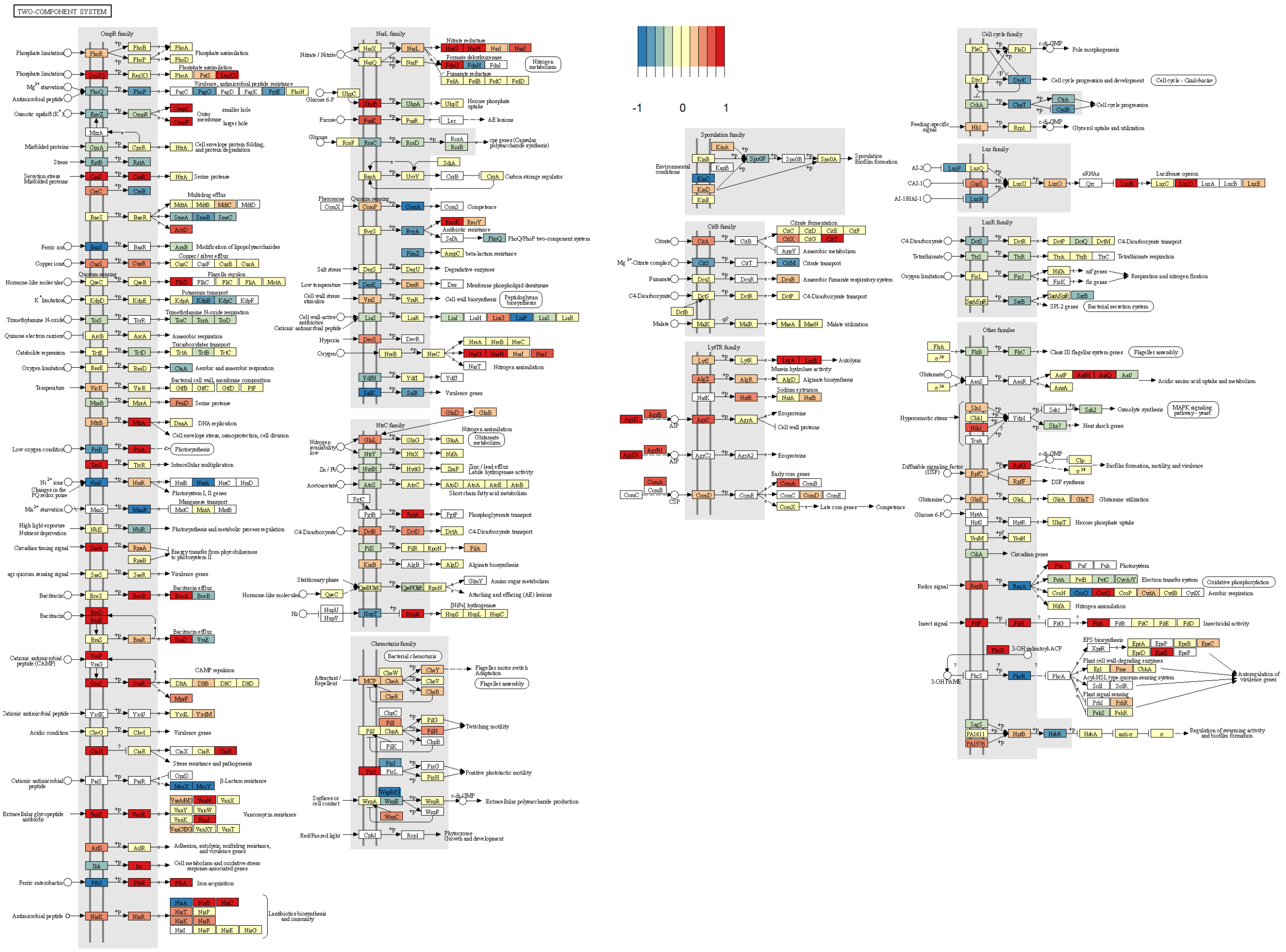

Figure S13. Representation of the metagenomic gene abundance data for two-component system pathway (ko02020). KO's (orthologs) more abundant among L-REI animals in red and genes more frequent in H-REI in blue, respectively. Values outside the range –1 to 1 truncated within these boundaries. KO's missing in the current data are white.

Table S3. Abundances of KEGG functional categories in L-REI and H-REI groups.

**Level 1**	**Level 2**	**L-REI**	**H-REI**
Cellular Processes	Cellular community - prokaryotes	0.017130364	0.015489674
Cellular Processes	Cell motility	0.00399166	0.004184261
Cellular Processes	Cell growth and death	0.026899723	0.030919793
Cellular Processes	Transport and catabolism	0.024845639	0.031490869
Cellular Processes	Cellular community - eukaryotes	0.009146255	0.012032616
Environmental Information Processing	Membrane transport	0.018809359	0.017593917
Environmental Information Processing	Signal transduction	0.069837588	0.086692054
Environmental Information Processing	Signaling molecules and interaction	0.000118248	0.000141923
Genetic Information Processing	Translation	0.02551893	0.025520267
Genetic Information Processing	Folding sorting and degradation	0.020256588	0.021273769
Genetic Information Processing	Transcription	0.004949323	0.005258751
Genetic Information Processing	Replication and repair	0.037823102	0.034519701
Metabolism	Carbohydrate metabolism	0.095035705	0.086173448
Metabolism	Lipid metabolism	0.017918707	0.016871767
Metabolism	Metabolism of cofactors and vitamins	0.025708803	0.022989969
Metabolism	Energy metabolism	0.032827458	0.029540102
Metabolism	Amino acid metabolism	0.050532389	0.044615054
Metabolism	Nucleotide metabolism	0.040765731	0.037261803
Metabolism	Biosynthesis of other secondary metabolites	0.01274835	0.011230431
Metabolism	Metabolism of terpenoids and polyketides	0.006241103	0.005555743
Metabolism	Xenobiotics biodegradation and metabolism	0.005986598	0.005549583
Metabolism	Metabolism of other amino acids	0.009966025	0.008931411
Metabolism	Glycan biosynthesis and metabolism	0.018371988	0.016713928
Metabolism	Global and overview maps	0.284146639	0.25430495
Organismal Systems	Endocrine system	0.047014076	0.058068726
Organismal Systems	Immune system	0.016006097	0.019950162
Organismal Systems	Aging	0.010179524	0.012309653
Organismal Systems	Circulatory system	0.006116842	0.008069772
Organismal Systems	Development and regeneration	0.002186447	0.002863468
Organismal Systems	Environmental adaptation	0.015005655	0.018684685
Organismal Systems	Nervous system	0.02004027	0.025012429
Organismal Systems	Sensory system	0.007566959	0.009912256

Organismal Systems	Excretory system	0.004106157	0.005282397
Organismal Systems	Digestive system	0.012201699	0.014990671
